# The emerging role of intestinal stem cells in ulcerative colitis

**DOI:** 10.3389/fmed.2025.1569328

**Published:** 2025-03-25

**Authors:** Siqing Chen, Zhang Qin, Sainan Zhou, Yin Xu, Ying Zhu

**Affiliations:** ^1^Department of Gastroenterology, The First Hospital of Hunan University of Chinese Medicine, Changsha, Hunan, China; ^2^The Fourth Hospital of Changsha (Changsha Hospital Affiliated with Hunan Normal University), Changsha, Hunan, China

**Keywords:** ulcerative colitis (UC), intestinal mucosal barrier, intestinal stem cells (ISCs), immune response, inflammation

## Abstract

Ulcerative colitis (UC) is a chronic idiopathic inflammatory disease affecting the colon and rectum. Characterized by recurrent attacks, UC is often resistant to traditional anti-inflammatory therapies, imposing significant physiological, psychological, and economic burdens on patients. In light of these challenges, innovative targeted therapies have become a new expectation for patients with UC. A crucial pathological feature of UC is the impairment of the intestinal mucosal barrier, which underlies aberrant immune responses and inflammation. Intestinal stem cells (ISCs), which differentiate into intestinal epithelial cells, play a central role in maintaining this barrier. Growing studies have proved that regulating the regeneration and differentiation of ISC is a promising approach to treating UC. Despite this progress, there is a dearth of comprehensive articles describing the role of ISCs in UC. This review focuses on the importance of ISCs in maintaining the intestinal mucosal barrier in UC and discusses the latest findings on ISC functions, markers, and their regulatory mechanisms. Key pathways involved in ISC regulation, including the Wnt, Notch, Hedgehog (HH), Hippo/Yap, and autophagy pathways, are explored in detail. Additionally, this review examines recent advances in ISC-targeted therapies for UC, such as natural or synthetic compounds, microbial preparations, traditional Chinese medicine (TCM) extracts and compounds, and transplantation therapy. This review aims to offer novel therapeutic insights and strategies for patients who have long struggled with UC.

## 1 Introduction

Ulcerative colitis (UC) is a chronic inflammatory disease of the colon and rectum, which attacks patients with UC recurrently and is still not cured for a long time when it is treated with conventional medications (1). The incidence of UC progressing to colorectal cancer has increased over the years; this progression is also a significant factor contributing to the mortality of patients with UC (2). Long-term recurrence of the condition not only diminishes the quality of life for patients with UC, but also presents a significant medical burden. Currently, the drugs used in the clinical treatment of UC, such as aminosalicylic acid, sulfasalazine, corticosteroid, immunosuppressants, and monoclonal antibodies, are used primarily to control the inflammatory reaction and excessive immune response to relieve the clinical manifestations. However, relapse, aggravation, drug resistance, and side effects may occur following drug withdrawal, presenting a core challenge for both patients and clinicians (3, 4).

The intestinal mucosal barrier comprises many kinds of intestinal epithelial cells, including secretory goblet cells and enteroendocrine cells. The loss of intestinal epithelial cells is a prominent pathological feature in developing inflammatory bowel disease (IBD), including UC (5). Intestinal epithelial cells are differentiated from intestinal stem cells (ISCs); thus, promoting the proliferation and differentiation of ISCs is crucial to repairing the intestinal mucosal barrier. At present, the etiology of UC is unclear. Still, with the progress of research in UC, it is gradually revealed that intestinal barrier dysfunction is closely related to the occurrence and development of UC. Additionally, ISCs play an essential role in maintaining the barrier integrity of intestinal mucosa. The mechanism of ISCs to maintain barrier function has become a hot issue for researchers. This review will focus on the relevant mechanisms by which ISCs are involved in the pathophysiology of UC, the latest evidence for ISC function and related markers, and their potential roles in therapeutic targets for UC.

## 2 Intestinal mucosal barrier and UC

### 2.1 Formation and function of intestinal mucosal barrier

The intestinal mucosal mechanical barrier, together with the chemical barrier, constitutes a significant part of the intestinal mucosal barrier. Furthermore, a healthy mucosal barrier effectively segregates the intestinal lumen and prevents the invasion of pathogenic antigens (6, 7). The mechanical barrier is the cornerstone of the intestinal barrier. It is a single-cell-dense inner layer of the intestinal with various epithelial cells with different functions, such as absorptive enterocytes and goblet cells (8). The chemical barrier comprises mucus secreted by the intestinal epithelium, digestive juice, and antibacterial substances produced by commensal microbes in the intestinal lumen (9). They act as guardians of the intestinal mucosal barrier, protecting against harmful substances that come into direct contact with intestinal epithelial cells.

Driven by the functional properties of the intestine, the intestinal epithelium under prolonged high-intensity mechanical stress and other environmental injuries can quickly trigger infection and wear of the epithelial base (10). For this reason, the lifespan of mature intestinal epithelial cells is very short, usually only a few days, which results in a high frequency of renewal (11). The renewal of intestinal epithelial cells is derived from crypts, where ISCs accumulate. It is because of the normal activity of ISCs that new intestinal epithelial cells are constantly produced (11). This cycle results in a solid and continuous intestinal barrier that can be repaired promptly if damaged. The renewal of intestinal epithelial cells follows a strict and complicated procedure. Once one link is affected, it will affect the integrity of intestinal epithelial cells and the normal function of the intestinal mucosal barrier, leading to a series of intestinal diseases, especially UC.

### 2.2 The role of intestinal mucosal barrier in UC

**FIGURE 1:**
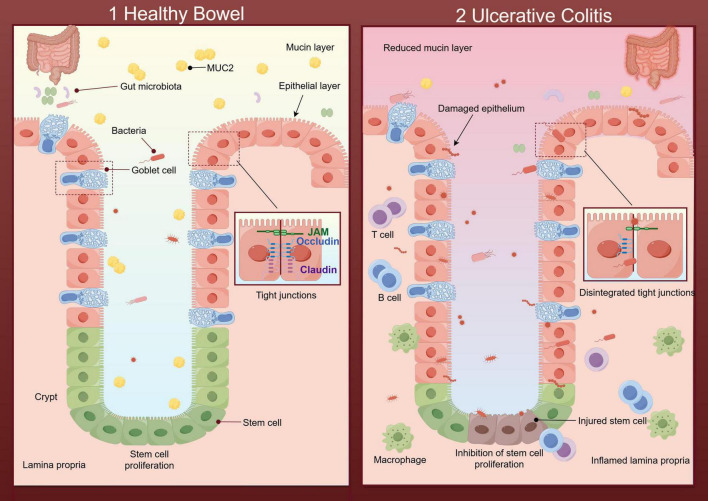
Diagram of the healthy intestinal barrier and its impairment during ulcerative colitis (UC) (By Figdraw). (1) Healthy bowel: The mucin layer protects the epithelial surface, helping to regulate the intestinal microbial environment and preventing harmful bacteria from directly contacting the epithelial layer. Tight junctions with defensive properties maintain the intact structure of the epithelial layer. The intestinal stem cells (ISCs) renew effectively within the epithelium. And there is no immune cell aggregation in the healthy lamina propria. (2) UC: The beneficial substances in the mucin layer are reduced, along with the imbalance of intestinal flora caused by the disruption of intestinal homeostasis. Destructive flora increases and further damages the epithelial layer through direct contact with it. In the epithelial layer, the regeneration of ISCs and the tight junctions are disintegrated. The inflamed lamina propria showed a large number of immune cells.

Impaired intestinal epithelial barrier function is one of the critical pathophysiological features of UC (12). Studies have shown intestinal mucosal barrier permeability increases in IBD due to severe injury of the mucin layer and the epithelial layer (13). The increased permeability makes it easier for invasive bacteria to penetrate damaged mucosa, triggering a range of immune responses. With abnormal immune responses, immune factors and inflammatory factors are produced in an unbalanced manner, beyond the ability of the body to regulate, which results in the aggravation of clinical inflammatory manifestations of UC (summarized in Figure 1).

Further studies have found that the intestinal mucosal barrier is a complex structure that allows the absorption of nutrients while protecting against intestinal pathogens and regulating immunity (14). Moreover, the normal functioning and repair of the intestinal mucosal barrier are multifactorial and dynamic processes, achieved through multiple defense mechanisms involving various cell types; these mechanisms create a protective barrier in the intestinal mucosa through combined efforts (15, 16). This flexible protective barrier can trigger an inflammatory cascade in response to stimuli related to various factors, such as pathogens and immune imbalance, and UC is a typical case (17). Currently, most treatments aim to reduce inflammation at sites of damaged mucosa, but these approaches fail to halt the inflammatory process or repair the intestinal barrier effectively.

In conclusion, the study of promoting the repair of the intestinal mucosal barrier is essentially to explore the mechanism of maintaining the normal renewal of intestinal epithelium. This allows for the multiple integrated defense lines of the intestinal mucosal barrier and is devoted to the reconstruction of the barrier function. In addition, it allows medication to promote healing of the intestinal mucosa more effectively. Therefore, the repair of the intestinal mucosal barrier is an indispensable step in the treatment of UC.

## 3 ISCs and repair of intestinal mucosal barrier in UC

Intestinal stem cells are adult stem cells with high proliferation and differentiation potential, comprising two significant ISC populations (11). One population consists of crypt-based columnar cells (CBCs), known as active cycling ISCs, which promote epithelial renewal to maintain intestinal homeostasis (18). Another group, responsible for tissue maintenance, is called reserve ISCs (rISCs); these cells are located at the base of the crypt around the +4 position and are also referred to as quiescent ISCs (19, 20). Quiescent ISCs cycle slowly, are resistant to injury, and can generate multiple cell types upon injury or loss of active cycling ISCs to compensate for intestinal regeneration (19, 21).

**FIGURE 2:**
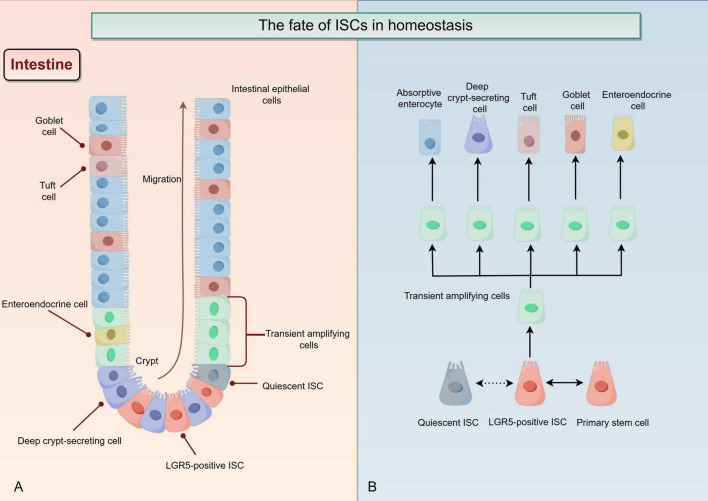
The fate of intestinal stem cells (ISCs) in homeostasis (By Figdraw). **(A)** In a homeostatic environment, the progeny of ISCs continuously migrate from the base of the crypt to the intestinal lumen, proliferate and differentiate into various types of cells, while stemness gradually decreases. The normal regeneration of ISCs ensures a constant replenishment of mature intestinal epithelial cells with extremely short lifespans. **(B)** LGR5-positive ISCs (LGR5^+^ ISC) and quiescent ISC have some mutual conversion modes. In a homeostatic environment, ISC divide asymmetrically, producing both primary stem cell and committed progenitor cell. The latter one then differentiated upward by stages of the transient amplifying intermediates into other terminally differentiated cells with specific functions and structures, including absorptive enterocytes, deep crypt-secreting cells, tuft cells, goblet cells, and enteroendocrine cells.

It is a continuous process that the progeny of ISCs migrates and differentiates from crypt base to intestinal lumen, and gradually moves toward aging and shedding (summarized in Figure 2). The intestinal stem cell moves into the intestinal lumen, proliferating and differentiating into various types of cells (22). With increasing degree of differentiation, the properties of the stem cell are gradually lost (23). Under external environmental stimuli, ISCs undergo asymmetric division into primary stem cells and committed progenitor cells; the latter then differentiate through transient amplifying intermediates into terminally differentiated cells with specific functions and structures, including absorptive enterocytes, deep crypt-secreting cells, tuft cells, goblet cells, and enteroendocrine cells, which collectively form the intestinal epithelium (summarized in Figure 2) (22, 24–26). Impaired intestinal barrier function is a significant aspect of UC pathology, leading to a series of abnormal immune-inflammatory responses (27, 28). The dysfunction of ISCs prevents the smooth and timely renewal of short-lived intestinal epithelial cells, hindering the repair of damaged colonic mucosa in UC (29, 30). One of the typical features of UC is the dysfunction of ISCs (31). It has been reported that the absence or reduction of DHX9 may affect the normal accumulation of R-loops, which in turn aggravates cGAS-mediated inflammation and genomic instability; these changes can damage the function of ISC and promote the development of IBD (32). It has also been reported that transplanting ISCs onto the damaged mucosa to facilitate intestinal barrier reconstruction may revolutionize treatment strategies in patients with UC (33). All of these studies highlight the crucial role of ISC in UC. Furthermore, different types of secretory cells differentiated from ISCs have distinct functions in the repair of damaged intestinal barrier in UC; for instance, goblet cells protecting damaged barriers from bacterial infection by secreting mucus, while tuft cells enhance prostaglandin E2-mediated epithelial repair to alleviate the response of chronic colitis to bacterial infection (34, 35). It has been demonstrated that colon tissue mucin2 (MUC2), a goblet cell marker protein, acts as a member of the mucin layer, preventing direct contact between harmful bacteria in the intestinal and colonic epithelial cells; for this reason, the reduction of MUC2 promotes mucosal and submucosal inflammation in the initiation and development of UC (9, 36). It has also been shown that the Leucine-rich repeat-containing G-protein coupled receptor 5 (LGR5) is a targeted gene for the Wnt signaling pathway, which serve as a major intestinal stem cell marker (37, 38). The process of normal proliferation and differentiation of LGR5-positive ISCs (LGR5^+^ ISCs) is indispensable for maintaining the integrity of intestinal epithelium and the proportion of various mature intestinal cells (39, 40). The key to rebuilding the intestinal mucosal barrier is to regulate the regeneration of ISCs and control the direction of their differentiation.

## 4 The marker proteins enriched in ISCs

The expression and distribution profile of ISC markers may be one of the mechanisms regulating the pathological and physiological processes of IBD and other intestinal diseases (41, 42). The marker is essential for identifying and cultivating ISCs. By detecting the expression of ISC markers and observing any changes, we can accurately assess their differentiation and activity levels, as well as investigate their heterogeneity and biological characteristics. This will aid in the treatment of intestinal diseases, the development of *in vitro* disease models, regenerative medicine, and drug development utilizing ISCs and intestinal organoids (43–45). Interestingly, the two different ISC populations of ISCs are not innate, and there seems to be a complex pattern of interconversion between them, both in the steady state and in response to injury (46, 47). The specific markers between them are not absolute, and there may be some mutual interaction. LGR5, OLFM4, SMOC2, ASCL2, and RNF43 are markers that are significantly enriched in active cycling ISCs. While, LRIG1, BMI1, TERT, HOPX, and MEX3A are more prevalent in quiescent ISCs. Notably, these proteins, distinct from other markers previously associated with ISCs, have been increasingly recognized in recent literature for their properties that contribute to the understanding and treatment of IBD.

### 4.1 Markers of active cycling ISCs

#### 4.1.1 LGR5

Leucine-rich repeat-containing G-protein coupled receptor 5 belongs to the rhodopsin-like family of G protein-coupled receptors and is expressed on the cell surface (48). LGR5 is found in a wide variety of organs, including the liver, ovaries, breast, and intestines (49). As one of the typical active cycling ISC populations, the intense proliferation and differentiation of LGR5^+^ ISCs contribute to the repair of intestinal mucosa injury (50). It has been reported that LGR5^+^ ISCs play an essential role in the occurrence and development of intestinal diseases, with UC being a prime example (51, 52). Furthermore, the Wnt and Notch signaling pathways jointly maintain the function of LGR5^+^ ISCs through some complex crosstalk, particularly in UC (53). Currently, various animal models are commonly employed to investigate the etiology and pathogenesis of UC and to evaluate the effectiveness of new drugs. Among these models are the dextran sodium sulfate (DSS) colitis model (54), the trinitrobenzene sulfonic acid (TNBS) colitis model (55), and the T cell transfer colitis model (56). The DSS colitis model is the most widely used due to its remarkable similarity to human with UC, including comparable symptoms and histological changes (54, 57, 58). Furthermore, LGR5^+^ ISCs in the colonic mucosa are particularly sensitive to injury induced by DSS (59). Ou et al. conducted an investigation into the intestinal tissue of patients with UC and in mouse models of colitis induced by DSS, as well as the isolation and three-dimensional culture of mouse intestinal crypts; they found that although Wnt and Notch signaling pathways co-regulate the proliferation and differentiation of LGR5^+^ ISCs, the balance between these signaling pathways may be disrupted in the context of IBD (52). This disruption can impede the normal regenerative processes of the damaged intestinal barrier. Recently, Tan et al. performed a study using the LGR5-2A-DTR model, which demonstrated that deletion of Lgr5-positive cells directly results in the loss of intestinal epithelium integrity *in vivo* and impedes organoid growth *in vitro* (60). This indicates that active cycling ISCs are crucial for the normal renewal of intestinal epithelial cells, and a sufficient quantity of LGR5^+^ ISCs contributes to the stabilization of the intestinal mucosal barrier. The self-renewal and high differentiation potential of LGR5^+^ ISCs determines their important role in intestinal mucosal lesions, especially in UC, which is characterized by pathological changes in colonic mucosa. The study on regulating LGR5^+^ ISCs-related targets and signaling pathways, as well as the transplantation of LGR5^+^ ISCs, will enhance the treatment of refractory UC.

#### 4.1.2 Olfm4

Olfactomedin-domain 4 (Olfm4) is a secretory glycoprotein in the Olfactomedin family, enriched in organelles of gastrointestinal tissues and bone marrow (61). Many studies showed that it has been used as a specific marker for active cycling ISCs in the human colon (62–65). Xing et al. observed the expression of IL-22-positive ILC3s in Olfm4 gene-deficient mouse model and patients with UC, revealing that Olfm4 could regulate the expression of IL-22-positive ILC3s, which was closely related to the immune homeostasis and inflammatory response in the colon; this study revealed the importance of Olfm4 in regulating colonic immunity and alleviating colonic inflammation (66). Olfm4 knockout mice are more susceptible to DSS-induced colitis than wild-type mice, showing a significant increase in colonic permeability and an exacerbation of the inflammatory response (67). Gersemann et al. conducted a study on colonic tissue from patients with IBD and control subjects, finding that Olfm4 protects the intestinal mucosa in IBD by binding to defensins in the intestinal mucus (64). These results suggest that OLFM4 may be a novel target for UC. In addition, studies have found that Olfm4 is enriched in polymorphonuclear myeloid-derived suppressor cells in colitis-associated intestinal tumor diseases, and the extent of its enrichment correlates with the degree of inflammation-to-tumor transition (68). Further study is needed to balance Olfm4 between UC and colitis-associated tumors.

#### 4.1.3 SMOC2

SPARC related modular calcium-binding protein 2 (SMOC2) is an extracellular glycoprotein expressed in many tissues and particularly enriched in LGR5-positive stem cells at the base of the intestinal crypt; these cells can be considered “omnipotent producers” capable of generating cells of any type in the crypts (69). Kim et al. observed a significant increase in the number of SMOC2-positive ISCs on day 11 of disease recovery from DSS-induced colitis mice (70). This result suggests that SMOC2-positive ISCs play a role in mucosal repair of colitis. Another study utilized a unique single-cell analysis system to culture and analyze intestinal organoids derived from mucosal biopsy specimens of patients with active Crohn’s disease; the findings revealed that the expression of several marker proteins enriched in ISCs was significantly increased, particularly SMOC2 (71). The researchers believe that the single-cell-level system they employed can be applied to a broader range of UC and Crohn’s disease organoids for further studies on the pathophysiology of ISCs, thereby advancing the understanding of IBD. These studies confirm the role of SMOC2 as a marker in inflammatory bowel tissue for ISCs. However, the specific relationship between SMOC2 and UC requires further investigation.

#### 4.1.4 ASCL2

Achaete-scute complex homolog 2 (ASCL2) is a protein that regulates the expression of various genes. It belongs to the basic helix-loop-helix family (72). Yi et al. found that ASCL2 upregulates the expression of IL-10, thereby attenuating the differentiation of pathogenic Th17 cells and ultimately promoting the recovery of IBD (73). Furthermore, they found that the expression of ASCL2 was strongly correlated with the behavior of the gut microbiota, which is a significant component of the intestinal barrier (73). These findings indicate a strong association between ASCL2 and the progression of IBD. The expression of ASCL2 is positively correlated with the population of LGR5^+^ ISCs; the decrease or disappearance of the former can cause a sudden drop in the latter, resulting in severe damage to the intestinal barrier (74). This suggests that ASCL2 may behave highly similarly to the classical marker protein LGR5 of ISCs in UC. As one of the critical targets of Wnt signaling, it is proposed to label ISCs by integrating the ASCL2 gene into human and mouse genomes through lentiviral transfection and organoid technology, and this provides a new approach to the treatment of related intestinal diseases (75). Taken together, maintaining the homeostasis of the intestinal mucosal barrier by regulating ISCs containing the stemness transcription factor ASCL2 may be a new therapeutic target for the treatment of IBD and warrants further investigation.

#### 4.1.5 RNF43

Ring finger protein 43 (RNF43), an E3 ubiquitin-protein ligase, is one of the typical ISC marker proteins (76, 77). RNF43 regulates Wnt signaling by promoting the turnover of the Wnt receptor, which allows ISCs to regenerate appropriately (78, 79). Additionally, RNF43 plays a role in maintaining the homeostasis of active cycling ISCs by ubiquitination Frizzled receptor and targeting it to the lysosomal pathway for degradation, ensuring the normal function of the intestinal mucosal barrier (80, 81). These suggest that RNF43, a marker protein for quiescent ISCs, may activate the regenerative behavior of active cycling ISCs to further promote the repair of the damaged intestinal barrier. Suntornsaratoon et al. found that DSS-induced colitis mice showed the same upregulation trend in the expression of Notch1 and RNF43 when exposed to methylnicotinamide; they proposed that the signaling pathway related to epithelial regeneration, which involves RNF43 and Notch, may be crucial for promoting the restoration of the intestinal barrier (82). This study highlights the significant role of RNF43 in facilitating epithelial regeneration in UC. However, mutations in the RNF43 gene override Wnt signaling to promote rapid proliferation of cells, leading to colitis-associated carcinogenesis, suggesting that aberrant RNF43 may be associated with colitis-associated carcinogenesis (83). As one of the special markers of active cycling ISCs, RNF43 is closely related to Wnt and Notch signaling. Proper regulation of RNF43 contributes to active cycling ISCs homeostasis, maintaining the daily renewal of healthy intestinal barrier epithelium and timely repair of the damaged intestinal barrier.

### 4.2 Markers of quiescent ISCs

#### 4.2.1 Lrig1

The Recombinant Leucine Rich Repeats And Immunoglobulin Like Domains Protein family comprises three proteins with different expression ranges and functions, of which, Recombinant Leucine Rich Repeats And Immunoglobulin Like Domains Protein 1 (Lrig1) is expressed in most tissues of the human body, particularly in the colon, the central organ involved in UC (84). The study found that Lrig1 was highly enriched in the small intestine and colon of mice, and the colon crypts of mice with Lrig1 deficiency were enlarged and hyperplastic (85). Similar studies have shown that the expression of Lrig1 rapidly increases in the colon after acute colonic epithelial injury, promoting cell proliferation and division to advance colonic crypt regeneration (86, 87). Using a novel induced knockout of Lrig1 mice, Hopton et al. found that Lrig1 is expressed in multiple tissues before birth in mice, while Lrig1 in the colon is essential for the growth of colonic epithelium (88). These studies are undoubtedly a theoretical affirmation of the contribution of LRIG1 protein in the restoration process of the intestinal barrier in UC. In addition, Lrig1 is also vital in the regulation of immune function. On the one hand, Lrig1 converts CD4 T cells without immunosuppressive function into regulatory T cells, suppressing the immune response and eventually reducing autoimmune symptoms in T cell transfer colitis models (56). On the other hand, Lrig1 deficiency impairs the suppressive function of naïve T cells, allowing them to switch to other T cell subsets that do not possess immunosuppressive functions (56). These results suggest that the presence of Lrig1 plays a positive role in diseases characterized by abnormal immune responses, such as UC. Regulating Lrig1 to restore colonic crypt structure may be a new therapy for UC.

#### 4.2.2 Bmi1

B-cell-specific moloney murine leukemia virus integrin site 1 (Bmi1), belongs to the polycomb repressive complex 1 family, is a protein that inhibits transcription and maintains stem cell stemness (89). Early studies showed that Bmi1 is one of the markers of rISCs and that when LGR5^+^ ISCs are injured or disappear, Bmi1-positive ISCs (Bmi1^+^ ISCs) promptly promote the regenerative response after intestinal epithelial injury (47, 90, 91). Lin et al. found that benzophenones significantly down-regulate the expression of the secretory cell markers MUC2 and KI67, as well as the expression of ISC marker Bmi1, in the colonic tissues of mice (92). Here, the decrease of Bmi1 indicates an impairment of intestinal mucosal barrier function, which is the pathological basis of a series of intestinal inflammatory responses. However, a recent study suggests that Bmi1 expression is enhanced in the colonic mucosa of refractory IBD and is associated with the clinical course of refractory IBD (93). Furthermore, the recent study revealed that Krüppel-like factor 4 regulates the differentiation of Bmi1^+^ ISCs into goblet cells in the injured state to promote the repair of the intestinal mucosal barrier (93). These findings indicate that Bmi1^+^ ISCs may benefit the self-healing phase of the clinical course of refractive IBD. Further investigations of the ISCs marker Bmi1 is of significant importance in exploring ways to reduce damage to the intestinal mucosal barrier in UC and restore intestinal epithelial homeostasis.

#### 4.2.3 TERT

Telomerase reverse transcriptase (TERT) is a catalytic subunit of telomerase with reverse transcriptase function, and its deletion leads to morphological changes of telomeres and eventually to senescence or cell death, which may be related to the self-renewal of tissue stem cells (94). Recent studies have revealed that TERT has non-classical functions independent of telomerase activity, such as activating multiple pathways to promote cell proliferation and regulate apoptosis (95, 96). Montgomery et al. found that the expression of mouse TERT-positive cells signals a slow cycle of ISCs, and their lineage-tracing studies showed that the strong differentiation ability of TERT-positive ISCs promotes the repair process of intestinal mucosa after injury (97). This study of TERT as one of the markers of quiescent ISCs revealed the importance of the slow cycle of ISCs for the recovery from intestinal mucosal injury. Recently, Zhu et al. conducted a study on transgenic colitis mice that express the TERT gene (TertKI mice); they found that TertKI mice are more resistant to DSS-induced colonic damage compared to wild-type mice (98). This increased resistance was shown through the improvement of fecal symptoms and colonic histopathology in TertKI mice, as well as their ability to tolerate greater colonic shortening when exposed to the same dose of DSS (98). This study highlights the important role of Tert as a maker protein in ISCs in relation to colonic inflammatory diseases, such as UC. Further targeted Tert-positive ISCs regulation may be a new direction in treating UC.

#### 4.2.4 HOPX

As the smallest homologous domain protein, homeodomain-only protein homeobox (HOPX) has been identified as a key transcription factor regulating cell proliferation and differentiation in many cell types (99, 100). Although it is recognized as one of the significant markers of quiescent ISC, its mechanism in quiescent ISC needs further study (101, 102). Stewart et al. conducted an evaluation of recovery from intestinal barrier injury using both a porcine model of mesenteric vascular occlusion *in vivo* and intestinal organoids *in vitro* (103). They discovered that HOPX-enriched ISCs are the primary drivers of intestinal barrier recovery (103). This finding suggests that HOPX plays a crucial role in the regeneration of the intestinal epithelium following injury. It has also been reported that HOPX, as a marker protein of quiescent stem cells, promotes the recovery of chronic intestinal inflammation in IBD from the direction of restoring the intestinal barrier (104). HOPX protein may be a potential target for restoring the intestinal barrier in UC. Interestingly, HOPX-positive cells can give rise to active cycling ISCs and all mature intestinal epithelial cell lines, suggesting that there may be some close and complex switching relationship between active cycling and quiescent ISCs (99, 105). In conclusion, HOPX, as a specific marker of quiescent ISCs, may be a target for controlling ISC proliferation in the damaged intestinal tissue of IBD; its special relationship with active cycling ISCs and other mature intestinal epithelial cell lines also determines its importance.

#### 4.2.5 MEX3A

Mex-3 RNA binding family member a (MEX3A) is a protein that controls the expression of various genes in multiple tissues. It is primarily involved in post-transcriptional regulation and plays an important role in embryonic development, epithelial homeostasis and tumorigenesis (106). MEX3A is also a well-recognized marker protein for quiescent ISCs (107). Furthermore, MEX3A has been reported to regulate colonic epithelial homeostasis in IBD (108, 109). Pereira et al. conducted a genome-wide screening of mouse colon cells using a three-dimensional culture system and gene screening techniques; they found that MEX3A was highly enriched in the mouse colon and plays a beneficial role in maintaining colonic homeostasis (110). Additionally, the study observed that ZO-1, a component of the tight junctions in the intestinal epithelium, was significantly upregulated on day 2 of culture by stable transfection of the MEX3A Caco-2 cell line (110). This upregulation may be one of the mechanisms by which MEX3A helps mitigate IBD. The development of UC is associated with epithelial-intestinal barrier dysfunction and the exacerbation of innate and adaptive immune responses (111). It is important to alleviate the excessive immune response while promoting the repair of the intestinal barrier. Interestingly, MEX3A helps to resist innate immune responses in various diseases by targeting multiple signaling pathways (112, 113). This reveals the possibility that MEX3A is involved in counteracting excessive immune responses to alleviate UC, but current research on this topic remains to be further explored. Overall, the important role of MEX3A in maintaining colonic barrier homeostasis and its specific link to immune responses make it a potential novel target for UC.

## 5 The regulation and operation mechanism of ISCs in UC

### 5.1 Wnt pathway

The canonical Wnt signaling pathway initiates the Wnt gene program through the cross-linking of β-catenin with T cell factor (TCF) in the nucleus (114, 115). When the expression of TCF4 in intestinal cells is impaired or absent, proliferating cells in the intestinal crypts of mice are progressively reduced until they are completely depleted (116). The canonical Wnt signaling pathway is vital in cell proliferation, differentiation and stem cell stemness maintenance (117, 118). The non-canonical Wnt signaling pathway shares some upstream components with Wnt receptors but does not activate the gene program that β-catenin protein expression depends on (119). There is a common target, Disheveled (DVL), in both canonical and non-canonical Wnt signaling pathways. DVL is recruited to the plasma membrane for degradation of β-catenin-related complexes, with degradation products (β-catenin) involved and transferred to the nucleus, which initiates the canonical Wnt pathway by activating TCF (120, 121). However, DVL recruitment activates other non-canonical Wnt pathways, including the Wnt/Ca^2+^ pathway (122). Thus, the level of DVL may be a critical control point for all Wnt signaling pathways (123–125).

The Wnt signaling pathway is the primary regulatory mechanism for the regeneration and differentiation of ISCs in the intestinal epithelium (126, 127), which is crucial for the intestinal epithelial cells and plays an important role in repairing intestinal mucosal damage in UC (53). A recent study has found that pigment epithelium-derived factor (PEDF) can inhibit the activation of canonical Wnt signaling pathways, thereby preventing ISCs from over-proliferation to maintain intestinal homeostasis; conversely, the reduction or deletion of PEDF was detrimental to the restoration of intestinal damage homeostasis in DSS-induced colitis mice (128). The key to adjusting the degree of Wnt activation to perform different functions lies in adjusting the expression level of PEDF. In addition, the inhibition of the Wnt signaling pathway rescues the loss of goblet cells in the colonic tissues of DSS-induced colitis mice, contributing to the repair of the damaged intestinal epithelial barrier (129). Multiple studies have shown that inactivation of the Wnt pathway promotes the differentiation of ISCs into goblet cells, thereby promoting the repair of the colonic mucosa in UC (130, 131). In a study of DSS-induced colitis mice and inflammatory intestinal organoids, Wang et al. found that the Wnt signaling pathway increases the number of enterocytes, goblet cells, and enteroendocrine cells derived from ISC differentiation, thereby enhancing the recovery of the damaged intestinal epithelium (132). However, it has been reported that inactivation of the Wnt pathway worsens intestinal barrier dysfunction in mice with DSS-induced colitis (133). Uchiyama et al. reduced colonic damage in DSS-induced colitis mice by increasing the expression of Wnt-5a. Furthermore, the researchers examined 70 patients with UC and found that a decrease in Wnt-5a was linked to relapse (134). The Wnt pathway is the main pathway regulating ISC proliferation and self-renewal. However, how Wnt signaling of various cell types in the intestinal epithelium regulates the direction of proliferation and differentiation of ISCs to maintain intestinal epithelial homeostasis is an active area of research.

### 5.2 Notch pathway

Notch signaling in mammals consists of various Notch ligands, Notch receptors, nuclear binding proteins, and regulatory factors, which together maintain the homeostasis of the human brain, muscle, and intestine (135–138). Notch1 expression is abundant in the human intestine (136), and the number of Notch ligands and receptors at the membrane level determines the intensity of signal activation (135). Additionally, the geometric shape of cell-cell contact at the membrane level influences the efficiency of signal activation (139). In intestinal tumor diseases, β-catenin in the Wnt pathway acts downstream of Notch signaling and is involved in this process by activating the expression of hairy and enhancer of split 1 (Hes-1) (140–142). However, such cross-linking has not been found in the relevant studies of UC.

Notch signaling is bidirectional for intestinal homeostasis. On the one hand, the activation of classical Notch signaling promotes the expression of the target gene Hes1, inhibits the activity of atonal homolog 1 (ATOH1), and attenuates the differentiation of ISCs into secretory lineages; this results in the reduction of secretory proteins such as MUC2, which eventually leads to impaired intestinal mucosal barrier (143, 144). Inhibition of Notch signaling promotes an increase in MUC2 expression, which allows the mucin layer to be repaired, thereby alleviating UC (145). Interestingly, on the other hand, activation of Notch signaling promotes the differentiation and proliferation of ISCs, favoring the maintenance of ISC homeostasis and the recovery of IBD, including UC (145–147). Activation of Notch signaling increases the accumulation of Olfm4 in the cytoplasm as an ISC marker, thereby facilitating the repair of the damaged intestinal mucosa in UC (148). Goblet cells have been shown to play a protective role primarily in the early stages of colitis (34, 149). Research has indicated that the increase in goblet cells resulting from the inhibition of Notch signaling contributes to the recovery in the acute colitis mouse model, while the regeneration of ISCs promoted by the activation of Notch signaling is also crucial for recovery in the chronic colitis mouse model (150). These findings suggest that for IBD, the two different effects of Notch signaling may be related to the different stages of the disease.

Overall, we need to take advantage of Notch signaling’s two-way regulation of intestinal homeostasis. Regulating the Notch signaling in a timely and appropriate manner is important to promote restoring intestinal homeostasis in treating UC.

### 5.3 Hedgehog (hh) pathway

Hedgehog protein is a secreted protein widely distributed in the gastrointestinal tract, skin, lung and other tissues (11). It is crucial in regulating stem cell activity to influence embryonic development and tissue regeneration (152). There are three human genes coding for Hh, namely Indian Hedgehog, Desert Hedgehog and Sonic Hedgehog (Shh) (153, 154). The canonical Hh signaling pathway primarily functions by directly affecting the expression of ligands such as smoothened, while the non-canonical Hh signaling pathway exerts the corresponding response in other ways (155). The Hh signaling pathway is a key regulator of ISC behavior, exhibiting complex interactions with Wnt, Notch, and other signaling pathways (156). Inhibition of Hh signaling suppresses the proliferation of ISCs triggered by injury (157). In addition, an early study has shown that impaired or chronic inhibition of Hh protein is associated with intestinal inflammation and death in adult animals (158). The activation of the Hh signaling reduces the disease activity index and colon pathological injury caused by DSS in mice. Additionally, impairment of Hh signaling in the intestinal tissues of patients with IBD leads to increased inflammation in the damaged intestinal mucosa, negatively impacting the proliferation of intestinal cells. Interestingly, inhibition of Hh signaling alleviates acetic acid-induced UC in mice (159). Hh signaling is up-regulated in the injured colon tissue of patients with UC, and the application of Hh inhibitors helps alleviate the mucosal pathological changes observed in the colon tissue (160). Furthermore, patients with IBD have increased Shh expression *in vivo* during active intestinal inflammation; furthermore, adding Shh inhibitor to inflammatory CACO-2 cells *in vitro* decreases the inflammatory response and increases the expression of tight junction protein (161). These results suggest that the regulation of Hh pathway is closely related to the occurrence and development of UC. Considering the significance of Hh signaling regulation for ISCs in repairing the damaged intestinal mucosal barrier and restoring intestinal homeostasis, modulating Hh signaling may represent a new approach to treating UC (162, 163). However, the mechanism by which Hh signaling regulate ISC homeostasis in UC is very complex, and research on this topic is limited both domestically and internationally. Further investigation of the regulatory mechanism of Hh signaling on ISCs in UC has positive significance for restoring the epithelial barrier and relieving UC.

### 5.4 Hippo/YAP pathway

The Hippo pathway regulates nuclear rejection and inactivation of YAP and Tafazzin (TAZ) through phosphorylation and dephosphorylation of a range of kinases, such as Mercaptopyruvate Sulfurtransferase (MST) and large tumor suppressor kinase 1/2 (LATS1/2) (164, 165). It mainly participates in the process of cell proliferation, differentiation and apoptosis, especially in the fate of stem cells (166). It is important to highlight that the fetal-like transcriptional program activated by YAP/TAZ signaling plays a crucial role in the reprogramming of intestinal epithelial cells, particularly during UC *in vivo* (86, 109). Recently, Yui et al. examined the fate transitions in intestinal epithelial regeneration using DSS-induced colitis mice and intestinal organoids; their findings revealed that injured intestinal epithelial cells activate the FAK/SRC-YAP/TAZ axis by remodeling the extracellular matrix (86). This activation subsequently trigger the fetal-like transcriptional program, leading to dedifferentiation and restoration of stemness in these cells (86). Essentially, this process allows the damaged epithelium to be temporarily reprogrammed to a primitive state until the normal homeostatic microenvironment is reinstated. Stem cell-like fetal intestinal epithelial cells generated by YAP/TAZ signaling are distinct from ISCs but equally beneficial for repairing the impaired intestinal barrier in UC.

YAP is abundantly expressed in colon tissue, particularly in the cytoplasm of LGR5^+^ CBC cells (167). Inhibition of a series of core kinases of the Hippo pathway leads to hyperactivity of YAP, which causes uncontrolled proliferation and undifferentiation of ISCs (168). The Hippo pathway is a classic pathway for intestinal cancer, but it is less involved in IBD (169). However, recent studies have shown that the Hippo/YAP signaling pathway can promote the restoration of the intestinal mucosal barrier by regulating ISCs after intestinal epithelial injury, especially in IBD such as UC (170, 171). Si et al. conducted a study on DSS-induced colitis mouse model and found that the downregulation of Hippo protein expression allows ISCs in the colonic crypts to actively proliferate, thereby repairing the impaired colonic mucosal barrier (172). Tian et al. conducted a study on microRNA-31 (Mir31) knockout DSS-induced colitis mice model and found that Mir31 could regulate Wnt and Hippo signaling pathways and promote epithelial regeneration after injury in IBD (170). Nterma et al. observed that the expression of LATS1/2, a key molecule in the Hippo pathway, was significantly elevated in human mucosa with UC compared with the control mucosa; this may be the mechanism by which intestinal mucosal barrier recovery is hindered (171). Additionally, a study by Fallah et al. on the HT29 cell line with YAP1 knockout *in vitro* discovered that inactivating YAP1 significantly shifts ISCs toward goblet cells instead of proliferation (173). Phosphorylation of YAP inhibits the Wnt/β-catenin signaling pathway, thereby preventing stem cell regeneration (174). Interestingly, Gregorieff et al. demonstrated that YAP induces reprogramming of LGR5^+^ ISCs by suppressing Wnt signaling, likely due to the complex interactions between YAP and Wnt (175). It has also been found that PLA2G2A inhibits Wnt signaling in colon tissue by increasing YAP phosphorylation, significantly reducing the ability of ISCs to form organoids (176). A recent report indicated alterations in Hippo signaling during different stages of recovery from TNBS-induced colitis in rats (177). In the early stage of colitis recovery, the Hippo signaling pathway is inhibited, leading to the active proliferation of colonic epithelial cells in the crypts; in the late stage of colitis recovery, the Hippo signaling pathway is activated, inhibiting epithelial proliferation and promoting intestinal epithelial differentiation (177). These results suggest that Hippo signaling, whether activated or inhibited, has the potential to reconstitute the impaired intestinal mucosal barrier in UC from different directions. Furthermore, several studies found that Notch signaling normally acts downstream of Hippo/YAP pathway, suggesting that intestinal epithelial proliferation resulting from the original signaling can be suppressed by suppressing Notch signaling (178, 179). These suggest that the Hippo pathway is one of the major pathways regulating ISC behavior in UC and that there is some complex crosstalk between the Wnt signaling pathway and the Notch signaling pathway in terms of the mechanism regulating ISCs in colitis.

### 5.5 Autophagy

Autophagy leads to lysosome-mediated clearance and re-release of organelles and biomolecules, a process of cell catabolism that belongs to programmed cell death (180). Different from other regulatory pathways, the autophagy of ISCs significantly contributes to the onset and progression of UC by directly leading to their programmed cell death. Regulation of autophagy is an effective treatment for various diseases, especially gastrointestinal, immune and cardiovascular diseases (181). Autophagy is a primary function of quiescent ISCs (182). Interestingly, recent attention has been paid to the autophagy mechanism in UC. Both overactivation and deletion of autophagy can negatively affect the recovery process of damaged intestinal mucosa in individuals with UC. Numerous pieces of evidence indicate that abnormal autophagy is frequently involved in the initiation and progression of UC (183). When autophagy is affected, the activity of ISCs will be impaired, and the direction of differentiation will be hindered. Deletion of autophagy related protein 7 induces p53-mediated apoptosis of Lgr5^+^ ISCs, resulting in impaired repair capacity of the intestinal epithelium (184). In addition, increased autophagy in colonic tissues of DSS-induced colitis mice was associated with pathological colon damage (185, 186). However, Proper autophagy guarantees the normal renewal and differentiation of ISCs, which is very important to maintain the physiological function of the intestinal epithelium. It is worth mentioning that promoting autophagy of Lgr5^+^ ISC, on the other hand, restores its impaired regenerative capacity, thus accelerating the recovery of damaged intestinal epithelium in UC (187). The activation of autophagy by IL-22 can also increase the expression of occludin, a tight junction protein closely related to ISCs, which is beneficial to the recovery of DSS-induced UC in mice (188, 189). In addition, a recent study by Parham et al. showed that immune mapped protein 1 deletion in the intestinal epithelium regulates the autophagic activity of active cycling ISCs and quiescent ISCs, which affects the proliferation and metabolism of ISCs and the efficiency of organ formation (182). As a broad regulatory mechanism, autophagy affects the physiological function and pathological changes of ISCs through multiple pathways, affecting the intestinal mucosal barrier function in UC positively or negatively. Further researches are needed to make good use of this double-edged sword in the treatment of UC.

**FIGURE 3:**
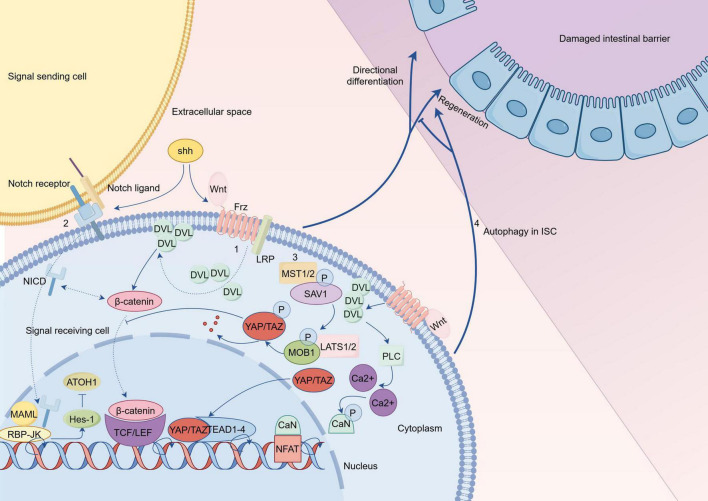
Hypothetical comprehensive regulatory mechanisms of intestinal stem cells (ISCs) in ulcerative colitis (UC) (By Figdraw). (1) The classical Wnt pathway is a trimer composed of Wnt proteins, frizzled receptors, and low density lipoprotein receptor (LRP) that recruits Disheveled (DVL) to the plasma membrane and degrades the β-catenin protein-related complex; subsequently, the accumulated β-catenin protein in the cytoplasm is transferred to the nucleus; finally, the active complex formed by β-catenin protein with T cell factor (TCF)/LEF protein initiates transcription of target genes. The Wnt/Ca^2+^ pathway is activated by DVL recruitment after binding of Wnt to Frz; this is followed by activation of PLC, which promotes the production of calcium ions; subsequently, calcium ions dephosphorylate CaN to act on NFAT in the nucleus, thereby initiating transcription of target genes. Complex interactions exist between Shh protein and Wnt signaling, as well as Notch signaling, which require further investigation. (2) In the Notch pathway, the cross-linking of Notch ligand and Notch receptor initiates the cleavage of Notch receptor and produces the cleavage product, the NICD; After NICD is transferred to the nucleus, it forms a binary complex with RBP-JK to initiate transcription of target genes; with MAML binding to this binary complex, expression of specific genes such as Hes is activated. (3) SAV1 binds to the kinase MST1/2 in the Hippo pathway to enhance kinase activity, and this complex subsequently phosphorylates the complex product of LATS1/2 with MOB1, up to which point YAP/TAZ is inactivated upon phosphorylation; phosphorylation of YAP inhibits Wnt signaling to hinder stem cell regeneration; this will lead to ISC differentiation into goblet cells rather than proliferation. When the upstream signal was blocked, the inactivated YAP/TAZ transferred to the nucleus and acted on transcription factors such as TEAD and TEF to initiate transcription of target genes. (4) Autophagy of ISCs affects the repair of damaged intestinal epithelium. Frz, frizzled; LEF, lymphoid enhancer factor; PLC, Phospholipase C; CaN, calcineurin; NFAT, nuclear factor of activated T-cells; NICD, Notch intracellular domain; RBP-JK, recombination signal binding protein-Jk; MAML, mastermind-like; SAV1, salvador homolog 1; MOB1, MOB kinase activator 1A; TEAD, transcriptional enhancer associated domain; TEF, transcriptional enhancer factor.

In summary, the fate of ISCs in UC is regulated by various pathways (as shown in Figure 3). The activation or silencing of specific targets enables proper regeneration and directed differentiation of impaired ISCs, thereby restoring the impaired intestinal mucosal barrier in UC, with the aim of treating it.

## 6 Targeted ISCs therapy for UC

The repair of the intestinal mucosal barrier is very important for the treatment of UC. Promoting the repair of the intestinal mucosal barrier essentially involves exploring the mechanism of maintaining the normal renewal of intestinal epithelium. ISCs regeneration and differentiation are beneficial to the restoration of intestinal mucosal barrier function in UC, and the targeted regulation of ISCs has become a hot research topic in the treatment of UC. Regulating the mechanism of ISCs in UC is expected to be an effective therapy for human with UC. Recent studies have identified some drugs that target and regulate ISCs to treat UC, including natural or synthetic compounds, microbial preparations, extracts and compounds of traditional Chinese medicine (TCM), among which transplantation therapy still needs more comprehensive experimental validation (Table 1).

**TABLE 1:** Summary of potential targeted intestinal stem cells (ISCs) therapeutics for ulcerative colitis (UC).

Category	Drug name	Model	Mechanism of action	References
Natural/synthetic compounds	SZN-1326-p	DSS-induced colitis mice	TNF-α↓, IL-6↓, IL-8↓, Lrig1↑, Lgr5↑, Rnf43↑, Axin2↑, Hes↑, ZO1↑	(190)
	Oat antimicrobial peptides	DSS-induced colitis rat	lgr5↑, β-catenin↑, YAP↑, cyclin D1↑	(197)
		Human colonic epithelial cells	TNF-α↓, IL-6↓, IL-1β↓, IL-4↑, IL-6↑
	OKGM peptosome-MIR31 microspheres	DSS-induced colitis mice	LATS1/2↓, STAT3↓, Ki67↑, YAP↑	(170)
Microbial preparations	LR	DSS-induced colitis mice	Lgr5↑,PCNA↑,Wnt3a↑,β-catenin↑	(209)
	L. reuteri D8	DSS-induced colitis mice	IL-1β↓, TNF-α↓; IL-22↑, PCNA↑, lysozyme↑	(210)
		TNFα-induced intestinal organoids	IL-22↑, lysozyme↑, Lgr5↑, Olfm4↑, Ascl2↑
	SYN	DSS-induced colitis mice	Wnt↓, Lgr5↓, cyclin↓, β-catenin↓, TNF-α↓, IL-1β↓,IFN-γ↓, IL-6↓, IL-10↑, occludin↑, claudin-1↑, ZO-1↑, LR↑, L. Murinus↑, AhR↑	(130)
Active extract of TCM	PF	DSS-induced colitis mice	TNF-α↓, IL-1β↓, Lgr5↑, SOX9↑, Ascl2↑, Muc2↑, Lysozym↑, Villin↑	(218)
		TNFα-induced colon organoids	Lgr5↑, SOX9↑, Ascl2↑, Muc2↑, Lysozym↑, Villin↑	
		LPS-induced IEC-6 cell	PARP1↓, BAX↓, Bcl-2↑	
	cDFPW1	DSS-induced colitis mice	Endotoxin↓, PCNA↑	(221)
		DSS-induced colon organoids	Lgr5↑, Olfm4↑, Ascl2↑, IL-22↑
Compound prescriptions of TCM	WMW	DSS-induced colitis mice	TNF-α↓, IL-6↓, caspase-3↓, LATS1/2↓, p-YAP↓, p-TAZ↓, Ki67↑, YAP↑, TAZ↑, TEAD1↑, Lgr5↑, Axin2↑, Mucin 2↑, cyclin D1↑	(225)

### 6.1 Natural or synthetic compounds

Wnt mimetic is an artificial synthetic substance that mimics the process of Wnt signaling and has been shown to be widely used in treating various disease states, including intestinal inflammation (190–192). Unfortunately, the difficulty of purifying Wnt and the lack of receptor selectivity make the development of novel Wnt mimics a considerable effort (193). A recent study found that a novel Wnt mimic, SZN-1326-p, promotes ISCs regeneration and differentiation in a favorable direction by upregulating key markers of the Wnt pathway, including LGR5 and Rnf43, and rapidly repair DSS-induced damage of colonic epithelium; in addition, this study showed that the use of only one Wnt mimic did not have a trend toward intestinal hyperproliferation (190). Wnt mimics are safe potential therapeutic targets for human UC. Further studies on SZN-1326-P and other Wnt mimics are expected to be refined to make an early contribution to human with UC.

In addition, Antimicrobial peptide (AMP) is a substance widely considered beneficial to the recovery of UC (194, 195). Oat antimicrobial peptides are a novel synthetic AMP-like substance that is more beneficial to the bioavailability of active peptides and has recently shown to treat colitis (196, 197). Wang et al. observed the effect of Oat antimicrobial peptides on DSS-induced rat colitis; they found that the Oat antimicrobial peptides activate Wnt pathway-related target proteins, promoting the regeneration and differentiation of ISCs, aiding in the repair of the damaged intestinal barrier in rats (197). The discovery of the role of Oat antimicrobial peptides in promoting ISCs regeneration in UC is undoubtedly a significant contribution to the healing process of colitis.

microRNA-31 has been shown to enhance stem cell regenerative capacity by amplifying Wnt signaling, promoting the repair of the damaged intestinal mucosa in colitis (198). A study by Keith et al. showed that low expression levels of Mir31 in the colon of adult patients with IBD requiring surgery were associated with disease exacerbation; furthermore, inhibition of Mir31 expression in the colon of children with IBD is associated with poor disease progression (199). Furthermore, Mir31 expression first increases during disease recovery in patients with active UC and then decreases to normal levels near the end point of disease recovery (170). However, the treatment of IBD with Mir31 requires a mediator that allows it to act directly on the colon and rectum. Tian et al. showed that enema with a synthetic combination of oxidized konjac glucomannan (OKGM) peptosome-Mir31 microspheres promotes the recovery of body weight and colon length in DSS-induced colitis mice and reduces the colonic inflammatory response; furthermore, this new preparation regulates the Hippo signaling pathway, which activates the regeneration of dormant ISCs, contributing to these positive outcomes (170). It is worth mentioning that in DSS colitis mice with Mir31 knockout, the regeneration of ISCs in colonic tissue is limited, the recovery of the colonic epithelial barrier is slow, and the immune and inflammatory responses are more robust compared to wild-type mice (170). The development of OKGM peptosome-MIR31 microspheres for enema administration may represent a novel therapeutic option for humans with UC.

### 6.2 Microbial preparations

Most patients with UC experience long-term intestinal and extraintestinal symptoms, leading to significant psychological and physiological stress (200, 201). Studies have shown that microbial metabolism regulates the behavior and the fate of ISCs through Gut-Brain Axis (202, 203). In recent years, there has been increasing evidence that intestinal epithelium cells are constantly exposed to various types of microorganisms, and moreover, each type of cell has specific ways of detecting and responding to complex signals from these microorganisms (204–206). The fate of ISCs and the behavior of colonic crypts are closely linked to these microbial signals (205, 206). Intestinal microflora causes ISCs to proliferate and differentiate precisely through various pathways, such as immunity and metabolism, to maintain the integrity of the intestinal mucosal barrier function (207). Unfortunately, there is currently limited research on targeted ISCs-based microbial agents that can be utilized for treating UC.

A study demonstrated that Lactobacillus reuteri (LR) supports the maintenance of impaired ISC stemness under stress conditions through the glycolysis metabolic pathway (208). This finding suggests that LR may play a therapeutic role in UC by improving the regeneration of ISCs. Recently, Luo et al. discovered that treatment with LR improves weight loss and pathological colon damage in mice with DSS-induced colitis (209). Additionally, they found that LR increases the expression of the ISC marker protein LGR5 and the proliferating protein PCNA in epithelial cells in a dose-dependent manner (209). Furthermore, the classical ISC regeneration pathway, known as Wnt signaling, is significantly activated in colon tissue following LR treatment (209). Overall, the researchers propose that LR promotes ISC regeneration by activating the Wnt pathway, which helps restore the damaged intestinal barrier and alleviates the symptoms of colitis. The potential of LR to target and modulate ISC offers new hope for reconstructing the UC intestinal barrier. Hou et al. discovered that Lactobacillus reuteri D8 (L. reuteri D8) effectively colonizes the injured colonic mucosa in mice with DSS-induced colitis (210). Additionally, they found that L. reuteri D8 activates IL-22 in both colitis mice and intestinal organoidse; this activation promotes the regeneration of ISCs and effectively restores the intestinal mucosal barrier (210). The ability of L. reuteri D8 to colonize damaged colonic tissue and initiate ISC regeneration for mucosal repair suggests that it could be a potentially effective and safe antimicrobial agent against UC.

Mannan-oligosaccharide has been shown to regulate microbial abundance and improve the stability of the intestinal epithelial structure, which is beneficial in repairing the damaged intestinal mucosal barrier (130, 211). The synbiotic combination of L. paracasei VL8 and prebiotic mannan-oligosaccharide, known as SYN, increases the population of lactic acid bacteria in the intestine and promotes tryptophan metabolism-related goblet cells differentiation by enhancing the abundance of LR (130, 212). Additionally, SYN inhibits the overactivation of Wnt signaling, which prevents ISCs from excessive proliferation and promotes their differentiation into secretory cells, thereby rebuilding the intestinal mucosal barrier (130). Targeting ISCs by SYN to enhance the proliferation of secretory line cells restores the mucin layer of the intestinal mucosal barrier.

In conclusion, microbial metabolism represents a promising approach to modulate the function of ISCs for the treatment of UC. However, the challenge of enhancing the accuracy of microbial colonization and its signaling to effectively treat UC remains substantial.

### 6.3 Active extract of TCM

The active ingredient in TCM is a natural compound with medicinal properties that regulates the immune response and intestinal flora, while also repairing the intestinal mucosal barrier in cases of UC (213–215). Thus, the natural compounds derived from TCM are effective targets for treating UC.

Paeoniflorin (PF), a natural active extract of paeonia lactiflora, has therapeutic effects on inflammatory, tumor, and immune diseases (216, 217). It is worth mentioning that PF is a beneficial natural remedy for treating UC disease. In a study of DSS-induced colitis in mice combined with inflammatory organoids, Ma et al. found that PF improves the pathological damage of colon tissue in a dose-dependent manner (218). Interestingly, the researchers found that this therapeutic effect of PF benefits from the ability to reverse the reduced expression of ISC markers, LGR5 and ASCL2, and promote the differentiation of ISC into secretory lineages in damaged colon tissue (218). This ultimately helps reconstruct the damaged colonic mucosal barrier. Moreover, this study indicates that autophagy, mediated by PI3K-AKT signaling, may be the mechanism through which PF regulates ISC behavior (218). In conclusion, PF has the potential to improve the pathological tissue and molecular changes of UC by regulating the regeneration and differentiation of ISCs, ultimately leading to the treatment of UC.

Dendrobium fimbriatum Hook polysaccharide (CDFPW1) represents a significant natural active constituent within the Dendrobium species (219). This active extract has been shown to mitigate mucosal damage, inflammatory infiltration, and intestinal dysbiosis, which are hallmark features associated with IBD (220). Wang et al. demonstrated that CDFPW1 alleviates the pathological damage of the colon in DSS-induced colitis mice, increases the expression of typical marker proteins of ISCs, and promotes the proliferation of intestinal epithelial cells associated with ISCs (221). The effect of CDFPW1 on UC may be related to the behavior of ISC regeneration. In addition, this active extract of TCM enhances the secretion of IL-22 by intestinal lamina propria lymphocytes (221). However, the relationship between IL-22 and ISCs requires further investigation. It is imperative to highlight that oral CDFPW1 does not undergo degradation within the upper gastrointestinal tract; instead, it exerts its therapeutic effects directly upon the colonic mucosa (221). This characteristic significantly enhances the feasibility of using oral CDFPW1 as a treatment modality for UC.

### 6.4 Compound prescriptions of TCM

Compound prescriptions of TCM involve using a combination of different types of Chinese Medicinal Herbs based on their specific roles within the prescription-such as the monarch, the minister, the assistant, and the messenger. Each of them in the combination works together to achieve effects that may be challenging to attain with just one alone. This concept belongs to TCM theory, which aims to balance each Chinese Medicinal Herb more carefully to accentuate its strengths and reduce its side effects. Recent studies have demonstrated that various Chinese herbal compounds, including Wu-Mei-Wan (WMW) and Qingbai Decoction, significantly alleviate both the clinical and pathological symptoms of colitis in mice (222–226). This confirms the therapeutic effectiveness of these Chinese herbal compounds in treating UC. Unfortunately, research on compound prescriptions of TCM targeting ISC for treating UC is very limited. Recently, Yan et al. evaluated the efficacy of WMW therapy in mice with DSS-induced colitis and analyzed the resulting changes in the expression of relevant target proteins; they found that WMW activates the Hippo/Yap pathway, which promotes ISC regeneration to repair the damaged colonic mucosal barrier rapidly (225). Notably, these recovery effects were found to be dose-dependent. Overall, WMW is an effective therapeutic agent for UC, improving both symptoms and molecular changes associated with the condition.

### 6.5 Transplantation therapy

Disruptions in the repair mechanisms of the intestinal barrier, stemming from abnormalities in ISC renewal and differentiation, are closely associated with the occurrence and development of IBD. In recent decades, there has been an increase in foundational research investigating stem cell-derived organoids transplantation as a potential therapeutic approach for IBD (227–229). ISC-derived organoids repair damaged intestinal mucosa and more accurately replicate the genetic lineage of human intestinal epithelial cells in mice (230). This indicates that transplanting ISC-derived organoids may offer a new treatment for UC related to intestinal mucosal injury. Unfortunately, there are few basic experiments on ISC-derived organoids transplantation to treat intestinal diseases. One early report indicated that ISC-derived organoids transplanted into the injured colon remodel the damaged intestinal epithelium (231). Recently, Sugimoto et al. pioneered an innovative orthotopic xenograft model utilizing human normal colon organoids (232). In their study, LGR5^+^ ISCs derived from human colon tissue were cultured into organoids and subsequently transplanted into xenogeneic EDTA-injured colitis mouse mode; the results revealed that, despite the differences in stem cell turnover rates between humans and mice, the success rate exceeded 70% (232). Notably, the researchers did not observe any tumorigenic changes in the intestines of the recipient mice. These findings suggest that human colon LGR5^+^ ISCs has long-term genetic stability while preserving their capacity for proliferation and differentiation within xenogeneic contexts. Overall, for patients with UC who have experienced frequent recurrences or significant side effects with long-term use of traditional UC medications, ISC-derived organoids transplantation may be a truly effective method to rebuild the intestinal mucosal barrier. However, the *in vitro* culture and transplantation of ISC-derived organoids are major blows to patients suffering from the economic crisis of UC. Improving both basic and clinical research on the transplantation of LGR5-positive stem cell-derived organoids in patients with UC, while also reducing the costs associated with human ISC-derived organoids transplantation, is a critical issue in the field of digestive health. It is also important to note that while ISC-derived organoids transplantation for colitis represents a promising approach in regenerative medicine that may benefit patients with UC, its clinical application presents complex ethical issues. These issues include the source of the stem cells, the procedures involved, and the process of transforming the stem cells, all of which require careful consideration.

In conclusion, the targeted regulation of ISCs stemness is a new potential therapy for UC. The integrity of intestinal mucosal barrier function is the protective shield of intestinal epithelial cells against foreign harmful substances and endogenous inflammatory factors. Conventional medications primarily work by controlling inflammation and reducing excessive immune responses. In contrast, ISC therapies focus on enhancing stem cell properties at the source. This method repairs the damaged intestinal epithelial barrier, which is essential for preventing and treating UC. Although it is not fully understood how ISCs perceive and respond to various signals under both normal and pathological conditions, the animal experiments aimed at regulating the stemness of these cells to treat UC have not shown any adverse effects, including immune disorders or tumor formation (233). Targeted regulation of ISCs stemness remains the only truly effective approach to reconstructing the damaged intestinal epithelial barrier. Further molecular and clinical investigations to understand the modulation of ISC stemness for the treatment of UC are imperative. Researchers within the field of gastroenterology are placing significant emphasis on optimizing the application of targeted regulatory ISC therapeutics for the management of UC.

## 7 Conclusion and future perspectives

Currently, there is no consensus on the underlying causes of UC. However, the restoration of the intestinal mucosal barrier is impeded, which is a crucial factor underlying the clinical manifestations of UC. The imbalance of ISCs renewal and differentiation is the fundamental source of intestinal mucosal barrier restoration failure. Therefore, determining how to make ISCs renew and differentiate appropriately is an important way to explore the potential treatment for patients with UC. Understanding the regulatory and operational mechanisms of ISCs in UC can lead to the discovery of more effective treatments by modulating these cells. Additionally, identifying important markers of ISCs enhances this understanding. However, currently known clinical trials of involving ISCs-targeted therapeutics for UC treatment are not abundant, and in particular, ISCs transplantation techniques that directly reestablish intestinal mucosal barrier function have not been studied within patients with UC. Routine treatments fall short of meeting the needs of people who have been suffering from UC for a long time. It is essential to develop new therapies to reduce the recurrence rate and complications of UC while also considering the economic burden on patients. This review provides a theoretical basis for the targeted ISCs therapy. However, the targeted ISCs therapy for patients with UC requires further molecular experiments and more extensive clinical studies to expand its benefits.
